# Different Dietary Protein and PUFA Interventions Alter the Fatty Acid Concentrations, but Not the Meat Quality, of Porcine Muscle 

**DOI:** 10.3390/nu4091237

**Published:** 2012-09-05

**Authors:** Dirk Dannenberger, Karin Nuernberg, Gerd Nuernberg, Antje Priepke

**Affiliations:** 1 Research Unit of Muscle Biology and Growth and Genetics and Biometry, Leibniz Institute for Farm Animal Biology, Wilhelm-Stahl-Allee 2, 18196 Dummerstorf, Germany; Email: knuernbg@fbn-dummerstorf.de (K.N.); gnuernbg@fbn-dummerstorf.de (G.N.); 2 Institute of Animal Production, State Institute for Agriculture and Fishing Research, Wilhelm-Stahl-Allee 2, 18196 Dummerstorf, Germany; Email: a.priepke@lfa.mvnet.de

**Keywords:** reduced protein diet, linseed oil, sunflower oil, pig muscle, carcass traits, meat quality, fatty acids

## Abstract

The present study investigated the effect of a reduced protein diet in combination with different vegetable oils (sunflower seed oil or linseed oil) on carcass traits, meat quality and fatty acid profile in porcine muscle. Forty male Landrace pigs were allocated into four experimental groups (each *n* = 8) and one control group (*n* = 8) at a live weight of approximately 60 kg. The pigs were fed *ad libitum* from 60 kg to 100 kg live weight and restricted to 2.8 kg/day until they reached 120 kg. In contrast to other studies, the intramuscular fat content (IMF) did not increase in animals of groups fed a reduced protein diet and vegetable oils. The IMF ranged between 1.2% and 1.4%. The growth performance and meat quality of the *longissimus* muscle was not affected by the diet, but the average daily gain (ADG) and drip loss were affected. The muscle fatty acid concentrations were significantly affected by the diet, resulting in higher *n*-3 FA concentrations up to 113 mg/100 g muscle and lower *n*-6/*n*-3 PUFA ratio for pigs fed linseed oil-containing high- and reduced protein diets, compared to sunflower seed oil-containing diets.

## 1. Introduction

The manipulation of the fatty acid composition of farm animal adipose tissues has been of great interest in recent years due to an increasing demand for the production of meat with desirable nutritional and technological qualities [1]. In contrast, the risks associated with the consumption of red meat to human health (e.g., cancer, diabetes and coronary heart disease) are currently a controversial topic [2,3]. To reduce the risk of cancer, the World Cancer Research Fund report recommends limiting the consumption of red meat to less than 500 g per week [4]. The majority of evidence for the association of red meat with cancer shows an increase in cancer risk for consumers with the highest level of red meat consumption; however, the results of most studies have not reached the level of statistical significance [5].

Currently, the main strategies for altering the nutritional profile of meat are genetic selection and/or dietary manipulation. Selection for lean growth and against higher back fat thickness has been a major goal of the pig industry. Dietary strategies used to customize the fatty acid composition of pig fat have proven very effective because dietary fatty acids can be incorporated into pig muscle and adipose tissues with few modifications [6,7,8]. The intramuscular fat (IMF) content of the pork is one important trait that influences meat quality characteristics, such as meat tenderness, juiciness, flavor and taste [1]. The role of IMF is of particular interest in pigs because genetic selection for lean pigs has reduced its level to below 1% IMF, compared with 2%–4% IMF in pig muscle fifty years ago. Several studies have suggested a favorable relationship between IMF and meat tenderness and juiciness and recommended that a minimum level of IMF is needed to maximize meat tenderness and consequently increase consumer acceptance [1,9]. Selected monounsaturated fatty acids (MUFAs) and polyunsaturated fatty acids (PUFAs) have a number of health benefits. The majority of the health benefits have been associated with *n*-3 PUFAs [10]. Several studies have investigated the effects of dietary PUFA (18:3*n*-3, linolenic acid; 18:2*n*-6, linoleic acid) source, mainly linseed, rapeseed or sunflower supplements, on pig performance and the fatty acid composition of pig tissues. It is known that feeding a linseed diet increases the content of *n*-3 PUFA and decreases the *n*-6/*n*-3 PUFA ratio in all tissues, whereas feeding a sunflower seed diet leads to an increase in the *n*-6 PUFA contents [11,12]. An increase in the tissue *n*-3 PUFA has been observed under these conditions and has been correlated with the direct incorporation of dietary PUFA into pig tissues [7]. Animal nutrition has a major impact on fat quality and composition; for example, protein or lysine-deficient diets are known to increase the IMF contents in pigs. Several studies have shown that the IMF content in pigs can be increased without increasing back fat deposition by feeding pigs low protein diets during the growing or finishing phase. This might be due to a tissue-specific activation of the expression of lipogenic enzymes by the reduced protein diet [13,14,15]. 

The present study investigated the effects of feeding reduced protein diets in combination with different vegetable oils on carcass traits, meat quality and fatty acid profile in porcine muscle. To date, feeding reduced protein diets in combination with different vegetable oils to pigs has not been investigated. Based on the current knowledge, we hypothesized that the reduced protein diet in combination with linseed or sunflower seed oil would lead to an increase in the IMF content in porcine muscle without detrimental effects on meat quality.

## 2. Experimental Section

### 2.1. Experimental Design

Forty male Landrace pigs (castrates) were used in the diet experiment. The animals were allocated into five feeding groups (each *n* = 8) at a live weight of approximately 60 kg (Table 1). The pigs were fed *ad libitum* from 60 kg to 100 kg live weight and restricted to 2.8 kg/day until 120 kg. The animals in the experimental groups (1–4) were fed two different levels of protein and two different types of vegetable oil. The animals in the control group were fed a regular diet without plant oil supplementation. The chemical and fatty acid compositions of the diets of the five groups—high protein diet with sunflower seed oil (HPD-SO), high protein diet with linseed oil (HPD-LO), reduced protein diet with sunflower seed oil (RPD-SO), reduced protein diet with linseed oil (RPD-LO) and control (CON)—are presented in Table 2. The HPD was formulated to contain an average of 19.5% crude protein, and the RPD was formulated to contain an average of 15.5% crude protein. Sunflower seed oil (lipids high in linoleic acid, 18:2*n*-6) was used in the HPD-SO and RPD-SO diet groups, and linseed oil (lipids high in linolenic acid, 18:3*n*-3) was used in the HPD-LO and RPD-LO diet groups as fat sources (Table 2). The diets contained the same level of metabolizable energy (ME), approximately 13.6 MJ/kg. Thus, the energy/amino acid ratio was lower in the RPD groups. Acquisition of feed intake was performed for each single animal. The pigs were weighed once per week during the diet experiment. The pig experiment was carried out at the facilities of the University of Rostock, Faculty of Agricultural and Environmental Sciences. All experiments were conducted simultaneously to avoid seasonal effects between the different feeding groups. All diet composition components were analyzed before the beginning of the experiment as a basis for the final diet calculations. Diet samples were taken twice during the experiment and the chemical and fatty acid compositions were analyzed by a certificated laboratory (LUFA Rostock) according to VDLUFA guidelines. The results of the diet analyses are presented in Table 2. All animals were slaughtered at a target live weight of 120 kg in the abattoir of the Leibniz Institute for Farm Animal Biology in Dummerstorf (Germany). The slaughter and dressing procedures were performed in accordance with EU specifications. Immediately after slaughtering, tissue samples were collected from the right side of the carcass. *Longissimus* muscles used for the analysis of meat quality and fatty acid concentrations were taken from the 13th/14th rib and stored at −80 °C until analysis.

**Table 1:** Experimental designand dietcomposition of the feeding groups.

	Group 1 (HPD-SO)	Group 2 (HPD-LO)	Group 3 (RPD-SO)	Group 4 (RPD-LO)	Group 5 (CON)
Number (*n*)	8	8	8	8	8
Feeding	High protein diet with sunflower seed oil	High protein diet with linseed oil	Reduced protein diet with sunflower seed oil	Reduced protein diet with linseed oil	Control
Diet Composition *					
Barley	35.0	35.0	38.4	38.4	15.5
Wheat	26.5	26.5	36.0	36.0	26.5
Soybean meal	26.0	26.0	12.0	12.0	19.5
Rye	-	-	-	-	19.0
Maize	-	-	-	-	9.0
Fiber mixture	5.0	5.0	6.0	6.0	3.0
Fattening Supplement	3.0	3.0	3.0	3.0	7.5
Linseed oil	-	4.5	-	4.5	-
Sunflower oil	4.5	-	4.5	-	-
Lysine	-	-	0.1	0.1	-

* Percent based on original matter.

**Table 2:** Results of chemical- and fatty acid composition analyses of the diets.

	Group 1 (HPD-SO)	Group 2 (HPD-LO)	Group 3 (RPD-SO)	Group 4 (RPD-LO)	Group 5 (CON)
*Chemical Composition * *					
Dry matter	90.0	88.7	89.8	89.6	89.3
Crude protein	19.6	19.4	15.7	15.0	17.4
Crude fat	5.2	5.7	5.9	6.3	3.4
Crude fiber	4.7	4.6	5.3	5.5	4.2
Crude ash	4.9	4.9	4.8	5.0	4.8
Starch	36.9	36.2	40.0	40.7	40.1
ME (MJ/kg)	13.7	13.7	13.5	13.5	13.2
Amino Acids *					
Lysine	0.93	0.95	0.82	0.82	0.96
Methionine	0.26	0.27	0.22	0.22	0.24
Cysteine	0.31	0.31	0.26	0.27	0.30
Threonine	0.60	0.61	0.48	0.45	0.56
*Fatty Acids *^a^					
14:0	0.12	0.11	0.12	0.11	0.35
16:0	10.30	10.03	9.89	9.52	12.44
18:0	3.38	3.84	3.43	3.75	1.84
18:1*cis*-9	22.81	17.29	22.96	18.01	32.46
18:2*n*-6	52.94	29.43	51.69	31.77	39.07
18:3*n*-3	6.66	36.11	8.10	33.80	6.74

* Percent based on original matter; ^a^ proportion of total fatty acids.

### 2.2. Methods

#### 2.2.1. Meat Quality Parameters

The pH values of the *longissimus* muscle samples were measured at 45 min and 24 h *post mortem* by stabbing a pH-Star CPU (Matthäus, Ebenried, Germany) into the left carcass side at the 7th/9th rib. The color of the *longissimus* muscle samples was measured at 24 h using a Minolta CR 200 (Minolta GmbH, Ahrensburg, Germany) with triplicate measurements from a freshly cut surface using the parameters L* (brightness), a* (redness) and b* (yellowness). The drip loss procedure has been described previously [16]. For the determination of Warner-Bratzler shear force (WBSF), one section of *longissimus* muscle (2.54 cm) was packed in transparent foil 24 h after slaughter, and then cooked in a water bath at 80 °C for 1 h to an internal temperature of 70 °C. After cooling for 60 min at room temperature, 3–4 cores (12.7 mm) were cut from the steaks parallel to the muscle fiber orientation. The WBSF was measured with the Texture Analyser (Ahnsbeck) using a Warner-Bratzler blade (2.8 mm wide) [17]. The intramuscular fat content (IMF) of the *longissimus* muscle samples were analyzed using a FoodScan™ Meat Analyser (FOSS Analytic, Hillerod, Denmark). The measurements were based on near infrared (NIR) transmission and covered 16 measurement points. The results are the average of 16 measurements. The IMF contents were expressed as g/100 g muscle. 

#### 2.2.2. Fatty Acid Analysis

Samples of *M. longissimus* were thawed at 4 °C. After homogenization (Ultra Turrax, IKA, Staufen, Germany; T25, 3 × 15 s, 12,000 rpm) and the addition of the fatty acid C19:0 as an internal standard, the total lipids were extracted in duplicate using chloroform/methanol (2:1, v/v) at room temperature. The detailed sample preparation procedure has been described previously [7]. Briefly, all of the solvents contained of *tert*-butylhydroxytoluene (BHT) to prevent the oxidation of PUFAs. The final lipid extracts were redissolved in 300 µL of toluene, and a 25 mg aliquot was used for methyl ester preparation. Sodium methoxide in methanol was added to the samples, which were shaken in a 60 °C water bath for 10 min. Subsequently, 1 mL of 14% boron trifluoride (BF_3_) in methanol was added to the mixture, which was then shaken for an additional 10 min at 60 °C. Finally, the solvent containing the FAMEs was reduced to dryness under an oxygen-free nitrogen stream, and the FAMEs were resuspended in 100 µL of *n*-hexane and stored at −18 °C until use for gas chromatography (GC) analysis. The fatty acid analysis of the muscle lipids was performed using capillary GC with a CP-Sil 88 CB column (100 m × 0.25 mm, Chrompack-Varian, Lake Forest, CA, USA) that was installed in a PerkinElmer gas chromatograph Autosys XL with a flame ionization detector (PerkinElmer Instruments, Shelton, CT, USA). The detailed GC conditions were recently described [18]. Hydrogen was used as the carrier gas at a flow rate of 1 mL/min. The split ratio was 1:20, and the injector and detector were set at 260 °C and 280 °C, respectively.

#### 2.2.3. Statistical Analysis

The effects of the reduced protein diets and vegetables oils were estimated by one-way analysis of variance with fixed factor group (group 1 to 5) using the GLM procedure of the SAS software system (SAS^©^ Systems, Release 9.2, SAS Institute Inc., Cary, NC, USA). The least squares means (LSMs) and the standard errors (SEM) of the LSMs are given in the tables. All *post hoc* tests were performed at a significance level of *p* ≤ 0.05 using the Tukey-Kramer correction for multiple tests.

## 3. Results and Discussion

The results of growth performance and carcass trait measurements of male Landrace pigs are presented in Table 3. The average daily gain (ADG) was not affected by the experimental diets; however, the control group (CON) exhibited a lower ADG compared with experimental groups. The average daily feed intake (ADFI) was not affected by the diet. These results are consistent with recent studies investigating the effects of dietary protein level on growth performance and carcass traits in crossbred pigs [13,14]. Furthermore, the carcass traits, e.g., back fat, muscle area and liver and belly fat weights, were not influenced by the different experimental diets, except the hot carcass weight (Table 3). 

**Table 3:** Growth performance and carcass traits of Landrace pigs fed different diets.

	Group 1 (HPD-SO)	Group 2 (HPD-LO)	Group 3 (RPD-SO)	Group 4 (RPD-LO)	Group 5 (CON)	Signific.
	LSM_SEM_	LSM_SEM_	LSM_SEM_	LSM_SEM_	LSM_SEM_	
	( *n* = 8)	( *n* = 8)	( *n* = 8)	( *n* = 8)	( *n* = 8)	
Start weight (kg)	68.8_1.60_	69.7_1.60_	69.4_1.60_	69.0_1.60_	69.2_1.60_	0.997
Live weight at slaughter (kg)	120.6_0.75 _^a^	121.4_0.75 _^a^	123.2_0.75 _^a^	120.6_0.75 _^a^	117.8_0.75 _^b^	<0.001
ADG (g)	831.4_18.38 _^a^	835.2_18.30 _^a^	874.4_18.38 _^a^	840.6_18.38 _^a^	761.0_18.38 _^b^	<0.001
ADFI (g)	3135.6_49.25_	3195.6_49.25_	3191.4_49.25_	3195.0_49.25_	3214.2_49.25_	0.832
Hot carcass weight, left (kg)	49.0_0.58 _^a,b^	49.1_0.58 _^a,b^	50.5_0.58 _^a^	49.3_0.58 _^a,b^	47.7_0.58 _^b^	0.039
Hot carcass weight, right (kg)	47.9_0.38 _^a^	48.2_0.38 _^a^	48.9_0.38 _^a^	47.8_0.38 _^a^	46.4_0.38 _^b^	<0.001
Liver weight (kg)	1.7_0.04_	1.8_0.04_	1.7_0.04_	1.7_0.04_	1.8_0.04_	0.119
Belly weight (kg)	1.5_0.14_	1.7_0.14_	1.5_0.14_	1.4_0.14_	1.5_0.14_	0.544
Back fat (mm)	18.4_1.03_	19.3_1.03_	18.2_1.03_	19.2_1.03_	20.1_1.03_	0.710
Muscle area (cm^2^)	52.1_2.01_	50.3_2.01_	54.3_2.01_	51.1_2.01_	52.2_2.01_	0.692

Different small letters (^a^, ^b^) denote significant effect of diet groups (*p* ≤ 0.05); ADG: average daily gain; ADFI: average daily feed intake.

Selected meat quality parameters of the *longissimus* muscles of pigs are presented in Table 4. A number of studies have demonstrated that IMF and subcutaneous fat content might be manipulated independently through dietary means. For example, feeding a low protein diet increases the level of IMF with much smaller effects or no effects on subcutaneous fat content in pigs [9,13,14]. Inadequate dietary protein and/or lysine limit protein synthesis and increase the amount of energy available for fat deposition, resulting in higher IMF. In contrast, the IMF level in the Landrace pigs of the present study was not affected by the reduced protein level or by the use of linseed or sunflower seed oil in the diet. The IMF content ranged between 1.2% and 1.4% (Table 4). One possible explanation for unaffected IMF contents could be the smaller difference of protein level in the diets (19.4%–19.6% HPD) *vs.* 15.0%–15.7% in the RPD groups compared with other studies (10%–13% low protein *vs.* 18%–23% for the high protein diet) [13,14]. No significant effects were observed between the different diets for muscle color (L*, a*, and b*), consistent with the results of recent studies by Alonso *et al.* [9] and Guo *et al.* [14]. However, one other study detected higher L*, a* and b* color values in the *longissimus* muscles of pigs fed low protein diets due to higher IMF contents and higher concentrations of 12:0 and 14:0 in the muscle, which makes the IMF less translucent and leads to a greater color saturation [19]. Neither reduced protein level nor dietary vegetable oil supplementation affected shear force (WBSF), pH value or cooking loss of the *longissimus* muscle (Table 4). Our results indicate that the pigs of all diet groups were in good condition pre-slaughter, resulting in normal pH development with a tendency toward better water holding characteristics in the muscles of HPD-LO pigs (lower drip loss). The lack of significant differences in IMF contents between the dietary treatments can contribute to the similar shear forces measured in the *longissimus* muscle (Table 4). Additionally, comparable muscle shear force seems to be a reflection of a uniform growth and development pattern, indicating similar sizes of muscle fibers and IMF contents.

**Table 4:** Longissimus muscle quality of Landrace pigs fed different diets.

	Group 1 (HPD-SO)	Group 2 (HPD-LO)	Group 3 (RPD-SO)	Group 4 (RPD-LO)	Group 5 (CON)	Signific.
	LSM_SEM_	LSM_SEM_	LSM_SEM_	LSM_SEM_	LSM_SEM_	
	( *n* = 8)	( *n* = 8)	( *n* = 8)	( *n* = 8)	( *n* = 8)	
IMF (%)	1.3_0.17_	1.4_0.17_	1.4_0.17_	1.3_0.17_	1.2_0.17_	0.789
pH (45 min)	6.3_0.06_	6.4_0.06_	6.4_0.06_	6.4_0.06_	6.2_0.06_	0.078
pH (24 h)	5.4_0.02_	5.4_0.02_	5.4_0.03_	5.4_0.02_	5.4_0.03_	0.789
Color L*	47.7_0.56_	48.3_0.56_	47.1_0.56_	48.5_0.56_	47.6_0.56_	0.380
a*	7.6_0.32_	7.4_0.32_	7.4_0.32_	7.3_0.32_	7.7_0.32_	0.886
b*	1.6_0.23_	1.6_0.23_	1.2_0.23_	1.4_0.23_	1.1_0.23_	0.440
Drip loss (g/100 g)	3.5_0.41 _^a^	2.6_0.41 _^b^	2.8_0.41 _^a^	3.0_0.41 _^a^	4.6_0.41 _^b^	0.012
Cooking loss (g/100 g)	23.6_0.80_	21.0_0.80_	21.2_0.80_	21.9_0.80_	23.8_0.80_	0.440
Shear force(kg/cm^2^)	5.2_0.27_	4.7_0.27_	5.5_0.27_	4.9_0.27_	4.8_0.27_	0.189

Different small letters (^a^, ^b^) denote significant effect of diet groups (*p* ≤ 0.05); IMF: intramuscular fat.

**Figure 1:**
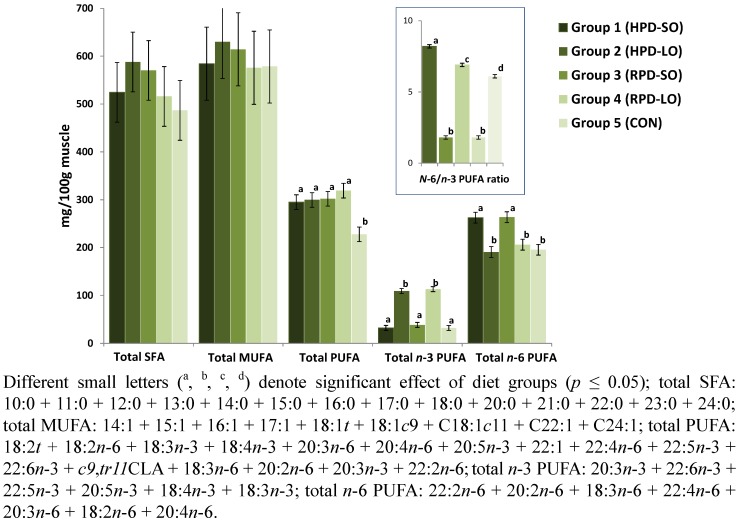
Total fatty acid concentrations (*longissimus *muscle) of Landrace pigs fed different diets.

The effects of dietary PUFA supplements, mainly linseed, rapeseed or sunflower seed oils, cake or seeds, on pig performance and fatty acid composition of tissues have been investigated intensively. It is known that feeding a linseed diet increases the *n*-3 PUFA content and decreases the *n*-6/*n*-3 fatty acid ratio in all tissues, whereas feeding sunflower seed leads to an increase in the *n*-6 PUFA contents due to the direct incorporation of the dietary PUFA into pig tissues [7,11,20,21]. The single fatty acid concentrations of the *longissimus* muscle in pigs are shown in Table 5, and the selected total fatty acid concentrations are presented in Figure 1. The muscle fatty acid concentrations of the present study appeared to be significantly affected by the diet, resulting in significantly higher *n*-3 FA concentrations (up to 113 mg/100 g muscle) in linseed oil-containing high- and reduced protein diets, compared to sunflower seed oil diets; only docosahexaenoic acid (22:6*n*-3, DHA) was not significantly increased. In most of the previous studies, the use of *n*-3 PUFA-rich vegetable oils showed no or only small effects on the intramuscular DHA level [7,15,21,22]. Feeding linseed oil-containing HPD- and RPD diets significantly decreased the concentration of single and total *n*-6 PUFA in *longissimus* muscle of pigs compared with the sunflower seed oil-containing fed groups and the control group (Table 5 and Figure 1). Consequently, the high level of *n*-3 PUFAs, which are beneficial for human nutrition, and the lower level of *n*-6 PUFAs in pigs fed linseed oil-containing diets caused a beneficially low *n*-6/*n*-3 PUFA ratio in the muscle compared with sunflower seed oil containing diets and the control diet. The *n*-6/*n*-3 PUFA ratio was 1.8:1 in muscle of pigs fed linseed oil containing HPD- and RPD diets, which corresponds to the *n*-6/*n*-3 PUFA ratio recommended by the German Nutrition Society (≤5:1) [23]. The concentrations of single saturated and total saturated fatty acids (SFA) were not affected by either the protein content or the type of oil supplement (Table 5 and Figure 1). Other studies showed that feeding a low protein diet with an increased IMF level resulted in elevated concentrations of 14:0, 16:0 and total SFAs, as a reflection of the higher IMF contents [19]. Additionally, oleic acid (18:1*cis*-9), the most abundant fatty acid in pig muscles, and total monounsaturated fatty acids (MUFAs) were not affected by the different dietary treatments.

**Table 5:** Fatty acid concentrations (longissimus muscle, mg/100 g muscle) of Landrace pigs fed different diets.

	Group 1 (HPD-SO)	Group 2 (HPD-LO)	Group 3 (RPD-SO)	Group 4 (RPD-LO)	Group 5 (CON)	Signific.
	LSM_SEM_	LSM_SEM_	LSM_SEM_	LSM_SEM_	LSM_SEM_	
	( *n* = 8)	( *n* = 8)	( *n* = 8)	( *n* = 8)	( *n* = 8)	
Sum FA	1403.4_150.4_	1517.3_150.4_	1486.3_150.4_	1410.6_150.4_	1292.9_150.4_	0.852
12:0	1.2_0.18_	1.4_0.18_	1.3_0.18_	1.2_0.18_	1.0_0.18_	0.633
14:0	16.8_2.76_	20.5_2.76_	19.2_2.76_	16.9_2.76_	15.1_2.76_	0.667
16:0	331.8_40.15_	368.2_40.15_	354.2_40.15_	321.0_40.15_	302.9_40.15_	0.792
16:1	38.7_6.45_	44.2_6.45_	41.4_6.45_	37.9_6.45_	37.6_6.45_	0.941
18:0	162.9_19.13_	183.5_19.13_	183.3_19.13_	164.3_19.13_	154.5_19.13_	0.761
18:1*cis*-9	475.3_61.02_	508.4_61.02_	500.6_61.02_	469.4_61.02_	467.4_61.02_	0.980
18:1*cis*-11	55.8_7.70_	60.7_7.70_	57.4_7.70_	53.2_7.70_	57.5_7.70_	0.975
18:2*n*-6	201.3_10.70_^a^	148.2_10.70_^b^	203.9_10.70_^a^	163.0_10.70_^a,b^	139.3_10.70_^b^	<0.001
18:3*n*-3	13.1_4.05_^a^	65.2_4.05_^b^	16.8_4.05_^a^	66.8_4.05_^b^	10.3_4.05_^a^	<0.001
20:2*n*-6	5.1_0.40_^a^	3.8_0.40_^a,b^	5.7_0.40_^a,c^	4.2_0.40_^a,b^	3.3_0.40_^b^	<0.001
20:3*n*-6	6.3_0.22_^a^	5.5_0.22_^a,b^	6.4_0.22_^a^	5.6_0.22_^a,b^	7.0_0.22_^a^	<0.001
20:3*n*-3	2.5_0.63_^a^	8.2_0.63_^b^	3.3_0.63_^a^	8.8_0.63_^b^	1.8_0.63_^a^	<0.001
20:4*n*-6	43.4_1.01_^a^	29.4_1.01_^b^	41.4_1.01_^a^	29.9_1.01_^b^	39.9_1.01_^a^	<0.001
20:5*n*-3	4.4_0.54_^a^	17.9_0.54_^b^	5.1_0.54_^a^	19.7_0.54_^b^	6.3_0.54_^a^	<0.001
22:4*n*-6	4.8_0.14_^a^	2.3_0.14_^b^	4.6_0.14_^a^	2.0_0.14_^b^	4.3_0.14_^a^	<0.001
22:5*n*-3	8.5_0.29_^a^	13.8_0.29_^b^	9.4_0.29_^a^	14.0_0.29_^b^	8.9 _0.29_^a^	<0.001
22:6*n*-3	3.7_0.23_^a^	3.5_0.23_^a^	3.6_0.23_^a^	3.6_0.23_^a^	4.7_0.23_^b^	0.005

Different small letters (^a^, ^b^) denote significant effect of diet groups (*p* ≤ 0.05).

## 4. Conclusions

In contrast to our hypothesis, a reduced protein diet in combination with linseed or sunflower oil did not lead to an increase in the IMF content of the *longissimus* muscle of male Landrace pigs. However, diets supplemented with linseed oil allow the production of pork meat with a high *n*-3 fatty acid content and low *n*-6/*n*-3 PUFA ratio, which is beneficial for human nutrition. Additionally, feeding pigs a reduced protein diet in combination with linseed or sunflower oil had no detrimental effects on muscle meat quality.
